# (Nitrito-κ^2^ *O*,*O*′)bis­[tris­(4-methyl­phen­yl)phosphane-κ*P*]silver(I)

**DOI:** 10.1107/S2414314622011488

**Published:** 2022-12-06

**Authors:** Frederick P. Malan, Kariska Potgieter, Reinout Meijboom

**Affiliations:** a Department of Chemistry, University of Pretoria, Lynnwood Road, Hatfield, Pretoria, 0002, South Africa; b Department of Chemical Sciences, University of Johannesburg, PO Box 524, Auckland Park, 2006, Johannesburg, South Africa; Vienna University of Technology, Austria

**Keywords:** crystal structure, silver(I) complex, *p*-tolyl phosphine

## Abstract

The mol­ecular structure of the title compound, [Ag(NO_2_)(C_21_H_21_P)_2_], exhibits a pseudo-tetra­hedral coordination around the central Ag^I^ atom. The compound crystallizes with one mol­ecule in the asymmetric unit in the monoclinic space group *P*2_1_/*n* with a rather long *b* axis [33.8752 (2) Å]. Weak C—H⋯O and C—H⋯N inter­actions consolidate the crystal packing. The nitrite-O atoms each occupy a single position in the coordination geometry.

## Abstract 2

The synthesis and single-crystal structure are described of a μ-NO_2_ silver(I) tris-*p*-tolyl­phosphine complex to be applied as an potential anti­cancer agent.

## Graphical abstract

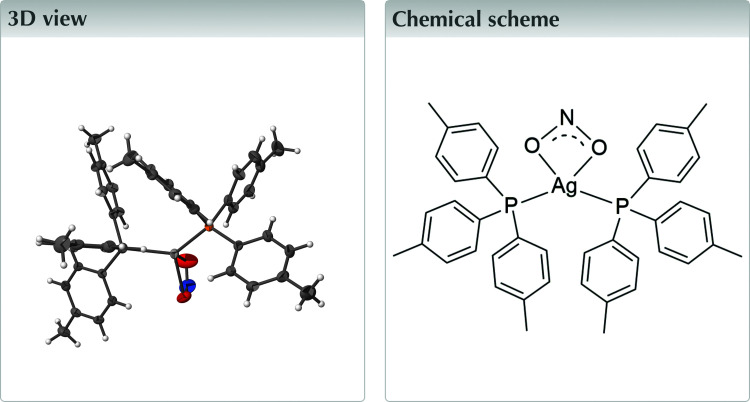

## Structure description

Silver is oligodynamic as a result of its excellent anti­microbial, anti­bacterial and anti­cancer properties (Meijboom *et al.*, 2009 ▸). Continuous development of phosphine silver(I) complexes has resulted in this class of compounds being evaluated against numerous cancer cell lines (Potgieter *et al.*, 2016 ▸). In this context, we report another phosphine silver(I) complex with nitrite as a co-ligand.

**Figure 1:**
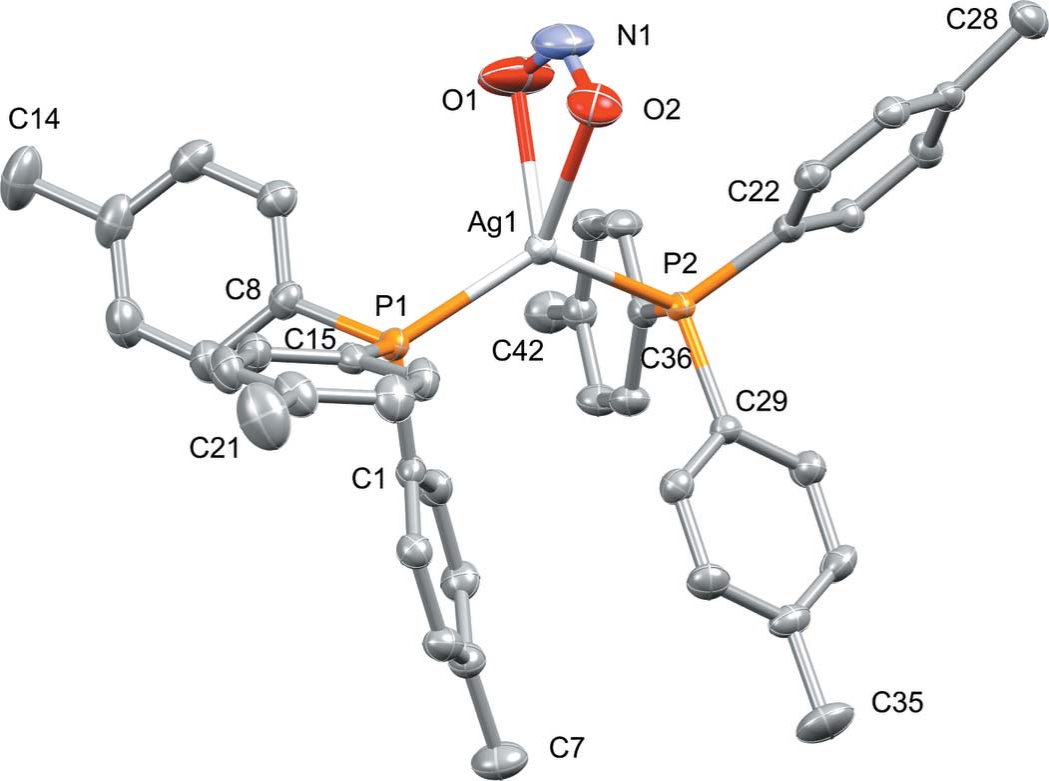
Perspective view of the mol­ecular structure of the title compound showing displacement ellipsoids at the 50% probability level. Hydrogen atoms are omitted for clarity.

The mol­ecular structure of the title compound is shown in Fig. 1 ▸. The asymmetric unit contains one complex mol­ecule, featuring a central Ag^I^ atom, two tris-*p*-tolyl­phosphine ligands, and one chelating nitrito ligand. Minor differences in the two Ag—P bond lengths are observed [Ag1—P1 = 2.4287 (5) Å; Ag1—P2 = 2.4570 (5) Å]. The nitrito ligand coordinates in a near symmetric fashion with similar bond lengths [Ag1—O1 = 2.4125 (19) Å; Ag1—O2 = 2.4227 (16) Å; N1—O1 = 1.249 (3) Å; N1—O2 = 1.233 (3) Å]. The pseudo-tetra­hedral coordination environment exhibited around the Ag^I^ atom stems from the three coordinating ligands, with corresponding bond angles of P1—Ag1—P2 [124.597 (16)°], P1—Ag1—O1 [116.26 (6)°], P1—Ag1—O2 [125.62 (4)°], P2—Ag1—O1 [107.68 (7)°], and P2—Ag1—O2 [107.83 (4)°]. The bidentate coordination of the nitrito ligand is underpinned by the O1—Ag1—O2 bite angle of 50.80 (7)°. The *ipso*-aryl carbon atoms of each of the phosphine ligands overlap in a near-staggered fashion when viewed down the P1—Ag1—P2 axis, presumably due to the steric effect of the bulky phosphine ligands. Corresponding torsion angles are P2—Ag1—P1—C1 = 9.90 (7)°, P2—Ag1—P1—C8 = −108.02 (8)°, P2—Ag1—P1—C15 = 128.73 (9)°, P1—Ag1—P2—C22 = −172.57 (7)°, P1—Ag1—P2—C36 = 70.75 (8)°, and P1—Ag1—P2—C29 = −47.35 (7)°. All of the aforementioned bond lengths and angles closely correspond to those of related Ag^I^ phosphine complexes (Meijboom *et al.*, 2009 ▸).

**Figure 2:**
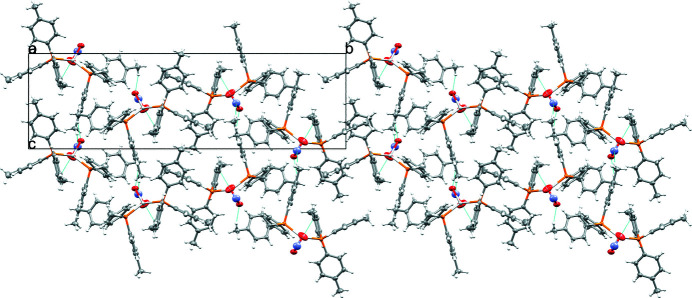
Packing diagram viewed along the *a* axis indicating two non-classical C—H⋯N and C—H⋯O hydrogen bonds as cyan dotted lines.

The complex packs in three dimensions as ribbons of isolated mol­ecular complexes. The mol­ecular packing is consolidated through weak inter­molecular C—H⋯O and C—H⋯N inter­actions (Fig. 2 ▸, Table 1 ▸) involving methyl donor groups and the N and O atom of the nitrito ligand as acceptor atoms; π-stacking inter­actions are not observed.

## Synthesis and crystallization

Tris-*p*-tolyl­phosphine (2 mmol) and silver nitrite (1 mmol) were dissolved separately in aceto­nitrile (10 ml). The two solutions were carefully mixed together and heated to 353 K for approximately 2 h. The solution was left to crystallize, and small clear colourless crystals were obtained.

## Refinement

Crystal data, data collection and structure refinement details are summarized in Table 2 ▸.

## Supplementary Material

Crystal structure: contains datablock(s) I. DOI: 10.1107/S2414314622011488/wm4174sup1.cif

Structure factors: contains datablock(s) I. DOI: 10.1107/S2414314622011488/wm4174Isup2.hkl

Supporting information file. DOI: 10.1107/S2414314622011488/wm4174Isup3.cdx

CCDC reference: 2223249

Additional supporting information:  crystallographic information; 3D view; checkCIF report

## Figures and Tables

**Table 1:** Hydrogen-bond geometry (Å, °)

*D*—H⋯*A*	*D*—H	H⋯*A*	*D*⋯*A*	*D*—H⋯*A*
C28—H28*B*⋯O2^i^	0.98	2.34	3.292 (3)	165
C42—H42*B*⋯N1^ii^	0.98	2.52	3.491 (4)	170

Symmetry codes: (i) 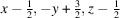 ; (ii) 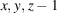 .

**Table 2:** Experimental details

Crystal data	
Chemical formula	[Ag(NO_2_)(C_21_H_21_P)_2_]
*M*_r_	762.57
Crystal system, space group	Monoclinic, *P*2_1_/*n*
Temperature (K)	150
*a*, *b*, *c* (Å)	10.8253 (1), 33.8752 (2), 11.3921 (1)
β (°)	116.880 (1)
*V* (Å^3^)	3726.22 (6)
*Z*	4
Radiation type	Cu *K*α
μ (mm^−1^)	5.43
Crystal size (mm)	0.21 × 0.15 × 0.12

Data collection	
Diffractometer	XtaLAB Synergy R, DW system, HyPix
Absorption correction	Multi-scan (*CrysAlis PRO*; Rigaku OD, 2022 ▸)
*T*_min_, *T*_max_	0.524, 1.000
No. of measured, independent and observed [*I* > 2σ(*I*)] reflections	46007, 7335, 7025
*R*_int_	0.037
(sin θ/λ)_max_ (Å^−1^)	0.617

Refinement	
*R*[*F* ^2^ > 2σ(*F* ^2^)], *wR*(*F* ^2^), *S*	0.026, 0.066, 1.03
No. of reflections	7335
No. of parameters	439
H-atom treatment	H-atom parameters constrained
Δρ_max_, Δρ_min_ (e Å^−3^)	0.45, −0.55

Computer programs: *CrysAlis PRO* (Rigaku OD, 2022 ▸), *SHELXT* (Sheldrick, 2015*a* ▸), *SHELXL* (Sheldrick, 2015*b* ▸) and *OLEX2* (Dolomanov *et al.*, 2009 ▸).

## full crystallographic data

### Crystal data

**Table:**

[Ag(NO_2_)(C_21_H_21_P)_2_]	*F*(000) = 1576
*M**_r_* = 762.57	*D*_x_ = 1.359 Mg m^−^^3^
Monoclinic, *P*2_1_/*n*	Cu *K*α radiation, λ = 1.54184 Å
*a* = 10.8253 (1) Å	Cell parameters from 37839 reflections
*b* = 33.8752 (2) Å	θ = 2.6–78.9°
*c* = 11.3921 (1) Å	µ = 5.43 mm^−^^1^
β = 116.880 (1)°	*T* = 150 K
*V* = 3726.22 (6) Å^3^	Block, colourless
*Z* = 4	0.21 × 0.15 × 0.12 mm

### Data collection

**Table:**

XtaLAB Synergy R, DW system, HyPix diffractometer	7335 independent reflections
Radiation source: Rotating-anode X-ray tube, Rigaku (Cu) X-ray Source	7025 reflections with *I* > 2σ(*I*)
Mirror monochromator	*R*_int_ = 0.037
Detector resolution: 10.0000 pixels mm^-1^	θ_max_ = 72.1°, θ_min_ = 2.6°
ω scans	*h* = −13→13
Absorption correction: multi-scan (CrysAlisPro; Rigaku OD, 2022)	*k* = −33→41
*T*_min_ = 0.524, *T*_max_ = 1.000	*l* = −13→14
46007 measured reflections	

### Refinement

**Table:**

Refinement on *F*^2^	Primary atom site location: dual
Least-squares matrix: full	Hydrogen site location: inferred from neighbouring sites
*R*[*F*^2^ > 2σ(*F*^2^)] = 0.026	H-atom parameters constrained
*wR*(*F*^2^) = 0.066	*w* = 1/[σ^2^(*F*_o_^2^) + (0.0325*P*)^2^ + 2.634*P*] where *P* = (*F*_o_^2^ + 2*F*_c_^2^)/3
*S* = 1.03	(Δ/σ)_max_ = 0.002
7335 reflections	Δρ_max_ = 0.45 e Å^−^^3^
439 parameters	Δρ_min_ = −0.55 e Å^−^^3^
0 restraints	

### Special details

**Table:**

Geometry. All esds (except the esd in the dihedral angle between two l.s. planes) are estimated using the full covariance matrix. The cell esds are taken into account individually in the estimation of esds in distances, angles and torsion angles; correlations between esds in cell parameters are only used when they are defined by crystal symmetry. An approximate (isotropic) treatment of cell esds is used for estimating esds involving l.s. planes.

### Fractional atomic coordinates and isotropic or equivalent isotropic displacement parameters (Å^2^)

**Table:**

	*x*	*y*	*z*	*U*_iso_*/*U*_eq_	
Ag1	0.65533 (2)	0.63978 (2)	0.42841 (2)	0.02180 (5)	
P1	0.77595 (5)	0.57779 (2)	0.44820 (5)	0.02163 (10)	
P2	0.65036 (5)	0.69483 (2)	0.28591 (4)	0.01930 (10)	
O2	0.57699 (19)	0.66458 (5)	0.58283 (16)	0.0448 (4)	
C29	0.81563 (18)	0.70936 (5)	0.29381 (18)	0.0199 (4)	
C2	1.0300 (2)	0.57988 (6)	0.43210 (18)	0.0244 (4)	
H2	1.0744	0.5726	0.5224	0.029*	
C25	0.44352 (19)	0.80859 (5)	0.34053 (19)	0.0235 (4)	
C1	0.8860 (2)	0.58141 (5)	0.36531 (18)	0.0220 (4)	
C36	0.54431 (19)	0.68106 (5)	0.11468 (18)	0.0210 (4)	
C26	0.52576 (19)	0.78451 (6)	0.44557 (19)	0.0244 (4)	
H26	0.5396	0.7912	0.5316	0.029*	
C22	0.57132 (18)	0.74079 (5)	0.30246 (18)	0.0204 (4)	
C37	0.5988 (2)	0.67381 (6)	0.02701 (19)	0.0252 (4)	
H37	0.6954	0.6768	0.0553	0.030*	
C27	0.58811 (19)	0.75088 (6)	0.42752 (18)	0.0228 (4)	
H27	0.6426	0.7346	0.5007	0.027*	
C15	0.8942 (2)	0.55952 (6)	0.61134 (18)	0.0228 (4)	
C23	0.49327 (19)	0.76552 (5)	0.19701 (18)	0.0223 (4)	
H23	0.4833	0.7596	0.1117	0.027*	
C3	1.1093 (2)	0.58891 (6)	0.3678 (2)	0.0281 (4)	
H3	1.2075	0.5882	0.4153	0.034*	
C8	0.6598 (2)	0.53656 (6)	0.37059 (19)	0.0255 (4)	
C24	0.42994 (19)	0.79888 (5)	0.21635 (19)	0.0245 (4)	
H24	0.3764	0.8154	0.1435	0.029*	
C40	0.3180 (2)	0.66502 (6)	−0.0583 (2)	0.0295 (4)	
H40	0.2212	0.6624	−0.0871	0.035*	
C39	0.3715 (2)	0.65788 (6)	−0.14628 (19)	0.0257 (4)	
C30	0.93294 (19)	0.68737 (6)	0.37074 (18)	0.0235 (4)	
H30	0.9262	0.6656	0.4202	0.028*	
C34	0.8276 (2)	0.74140 (6)	0.2224 (2)	0.0283 (4)	
H34	0.7484	0.7568	0.1699	0.034*	
O1	0.4291 (2)	0.63526 (8)	0.4201 (2)	0.0757 (7)	
C38	0.5131 (2)	0.66231 (6)	−0.10136 (19)	0.0279 (4)	
H38	0.5522	0.6574	−0.1597	0.033*	
C5	0.9032 (2)	0.59927 (6)	0.16815 (19)	0.0288 (4)	
H5	0.8589	0.6050	0.0767	0.035*	
C31	1.0598 (2)	0.69698 (6)	0.37583 (19)	0.0285 (4)	
H31	1.1391	0.6816	0.4282	0.034*	
C4	1.0474 (2)	0.59896 (6)	0.2356 (2)	0.0277 (4)	
C16	0.9020 (2)	0.52008 (6)	0.6477 (2)	0.0289 (4)	
H16	0.8397	0.5015	0.5877	0.035*	
C9	0.5328 (2)	0.53614 (6)	0.3729 (2)	0.0296 (4)	
H9	0.5054	0.5582	0.4072	0.035*	
C20	0.9859 (2)	0.58611 (6)	0.7025 (2)	0.0298 (4)	
H20	0.9801	0.6134	0.6810	0.036*	
C41	0.4026 (2)	0.67585 (6)	0.0705 (2)	0.0286 (4)	
H41	0.3637	0.6798	0.1295	0.034*	
N1	0.4549 (3)	0.65427 (8)	0.5226 (3)	0.0564 (6)	
C6	0.8232 (2)	0.59131 (6)	0.23182 (19)	0.0270 (4)	
H6	0.7251	0.5926	0.1845	0.032*	
C32	1.0722 (2)	0.72882 (6)	0.30523 (19)	0.0276 (4)	
C33	0.9545 (2)	0.75080 (6)	0.2280 (2)	0.0302 (4)	
H33	0.9614	0.7725	0.1786	0.036*	
C28	0.3653 (2)	0.84282 (6)	0.3593 (2)	0.0340 (5)	
H28A	0.3499	0.8380	0.4365	0.051*	
H28B	0.2759	0.8456	0.2811	0.051*	
H28C	0.4193	0.8671	0.3725	0.051*	
C19	1.0855 (2)	0.57326 (7)	0.8241 (2)	0.0352 (5)	
H19	1.1491	0.5917	0.8837	0.042*	
C17	0.9994 (2)	0.50771 (6)	0.7705 (2)	0.0349 (5)	
H17	1.0019	0.4807	0.7941	0.042*	
C18	1.0939 (2)	0.53389 (7)	0.8601 (2)	0.0353 (5)	
C10	0.4457 (2)	0.50374 (7)	0.3256 (2)	0.0355 (5)	
H10	0.3595	0.5038	0.3287	0.043*	
C13	0.6959 (2)	0.50441 (6)	0.3161 (2)	0.0363 (5)	
H13	0.7814	0.5045	0.3116	0.044*	
C11	0.4816 (2)	0.47134 (7)	0.2740 (2)	0.0381 (5)	
C42	0.2782 (2)	0.64668 (7)	−0.2869 (2)	0.0362 (5)	
H42A	0.2573	0.6184	−0.2918	0.054*	
H42B	0.3248	0.6525	−0.3414	0.054*	
H42C	0.1920	0.6618	−0.3191	0.054*	
C12	0.6074 (3)	0.47235 (7)	0.2686 (3)	0.0424 (6)	
H12	0.6332	0.4506	0.2316	0.051*	
C35	1.2110 (2)	0.73985 (9)	0.3151 (3)	0.0476 (6)	
H35A	1.2694	0.7508	0.4025	0.071*	
H35B	1.1987	0.7596	0.2478	0.071*	
H35C	1.2555	0.7163	0.3013	0.071*	
C7	1.1342 (3)	0.60951 (8)	0.1673 (2)	0.0437 (6)	
H7A	1.1357	0.6382	0.1581	0.066*	
H7B	1.0944	0.5973	0.0799	0.066*	
H7C	1.2289	0.5998	0.2194	0.066*	
C21	1.2034 (3)	0.51936 (9)	0.9912 (2)	0.0580 (8)	
H21A	1.2733	0.5400	1.0327	0.087*	
H21B	1.2477	0.4957	0.9779	0.087*	
H21C	1.1605	0.5129	1.0483	0.087*	
C14	0.3882 (3)	0.43554 (8)	0.2267 (3)	0.0555 (7)	
H14A	0.4229	0.4150	0.2944	0.083*	
H14B	0.3873	0.4255	0.1456	0.083*	
H14C	0.2940	0.4429	0.2095	0.083*	

### Atomic displacement parameters (Å^2^)

**Table:**

	*U*^11^	*U*^22^	*U*^33^	*U*^12^	*U*^13^	*U*^23^
Ag1	0.02209 (8)	0.02094 (8)	0.02489 (8)	0.00118 (5)	0.01282 (6)	0.00266 (5)
P1	0.0233 (2)	0.0191 (2)	0.0242 (2)	0.00116 (17)	0.01238 (19)	0.00274 (18)
P2	0.0196 (2)	0.0189 (2)	0.0222 (2)	0.00115 (17)	0.01191 (18)	0.00183 (17)
O2	0.0480 (10)	0.0589 (11)	0.0329 (8)	0.0042 (8)	0.0229 (8)	−0.0024 (8)
C29	0.0199 (9)	0.0200 (9)	0.0224 (9)	−0.0014 (7)	0.0118 (7)	−0.0016 (7)
C2	0.0266 (10)	0.0256 (9)	0.0213 (9)	0.0021 (8)	0.0111 (8)	0.0004 (7)
C25	0.0188 (9)	0.0212 (9)	0.0323 (10)	−0.0035 (7)	0.0132 (8)	−0.0059 (8)
C1	0.0275 (10)	0.0165 (8)	0.0245 (9)	−0.0008 (7)	0.0138 (8)	0.0010 (7)
C36	0.0220 (9)	0.0174 (8)	0.0257 (9)	0.0015 (7)	0.0127 (7)	0.0003 (7)
C26	0.0240 (9)	0.0279 (10)	0.0244 (9)	−0.0057 (8)	0.0136 (8)	−0.0070 (8)
C22	0.0191 (8)	0.0200 (8)	0.0246 (9)	−0.0016 (7)	0.0122 (7)	−0.0004 (7)
C37	0.0200 (9)	0.0309 (10)	0.0266 (9)	0.0010 (8)	0.0122 (8)	0.0008 (8)
C27	0.0208 (9)	0.0252 (9)	0.0217 (9)	−0.0007 (7)	0.0090 (7)	0.0013 (7)
C15	0.0266 (9)	0.0226 (9)	0.0242 (9)	0.0033 (7)	0.0159 (8)	0.0027 (7)
C23	0.0232 (9)	0.0227 (9)	0.0218 (9)	0.0002 (7)	0.0110 (7)	−0.0012 (7)
C3	0.0244 (10)	0.0321 (10)	0.0294 (10)	−0.0012 (8)	0.0135 (8)	−0.0037 (8)
C8	0.0257 (10)	0.0237 (9)	0.0247 (9)	−0.0002 (8)	0.0092 (8)	0.0050 (8)
C24	0.0215 (9)	0.0219 (9)	0.0268 (9)	0.0006 (7)	0.0080 (8)	0.0012 (8)
C40	0.0211 (9)	0.0313 (10)	0.0376 (11)	−0.0043 (8)	0.0146 (8)	−0.0090 (9)
C39	0.0263 (10)	0.0223 (9)	0.0271 (10)	0.0007 (8)	0.0109 (8)	0.0000 (8)
C30	0.0240 (9)	0.0236 (9)	0.0221 (9)	−0.0014 (7)	0.0097 (7)	0.0007 (7)
C34	0.0275 (10)	0.0262 (10)	0.0335 (10)	0.0028 (8)	0.0158 (9)	0.0065 (8)
O1	0.0377 (11)	0.115 (2)	0.0844 (16)	−0.0280 (11)	0.0367 (11)	−0.0450 (14)
C38	0.0269 (10)	0.0365 (11)	0.0251 (9)	0.0011 (8)	0.0161 (8)	−0.0007 (8)
C5	0.0337 (11)	0.0295 (10)	0.0233 (9)	0.0013 (8)	0.0129 (8)	0.0045 (8)
C31	0.0196 (9)	0.0351 (11)	0.0270 (10)	−0.0014 (8)	0.0072 (8)	−0.0012 (8)
C4	0.0323 (10)	0.0267 (10)	0.0288 (10)	−0.0008 (8)	0.0181 (9)	−0.0005 (8)
C16	0.0308 (10)	0.0232 (10)	0.0322 (10)	0.0007 (8)	0.0136 (9)	0.0020 (8)
C9	0.0288 (10)	0.0321 (11)	0.0273 (10)	−0.0008 (8)	0.0122 (8)	0.0022 (8)
C20	0.0424 (12)	0.0231 (9)	0.0270 (10)	0.0003 (9)	0.0184 (9)	0.0008 (8)
C41	0.0257 (10)	0.0333 (11)	0.0346 (11)	−0.0049 (8)	0.0204 (9)	−0.0093 (9)
N1	0.0489 (14)	0.0755 (16)	0.0647 (15)	0.0005 (12)	0.0433 (13)	−0.0034 (13)
C6	0.0251 (10)	0.0264 (10)	0.0278 (10)	0.0028 (8)	0.0105 (8)	0.0047 (8)
C32	0.0242 (10)	0.0360 (11)	0.0248 (9)	−0.0096 (8)	0.0131 (8)	−0.0076 (8)
C33	0.0334 (11)	0.0292 (10)	0.0325 (10)	−0.0077 (8)	0.0190 (9)	0.0020 (8)
C28	0.0311 (11)	0.0299 (11)	0.0424 (12)	0.0019 (9)	0.0178 (10)	−0.0084 (9)
C19	0.0447 (13)	0.0350 (11)	0.0239 (10)	−0.0020 (10)	0.0137 (9)	−0.0046 (9)
C17	0.0446 (13)	0.0241 (10)	0.0355 (11)	0.0077 (9)	0.0177 (10)	0.0082 (9)
C18	0.0436 (13)	0.0370 (12)	0.0246 (10)	0.0112 (10)	0.0148 (9)	0.0044 (9)
C10	0.0299 (11)	0.0410 (12)	0.0326 (11)	−0.0093 (9)	0.0113 (9)	0.0036 (9)
C13	0.0369 (12)	0.0287 (11)	0.0441 (13)	−0.0004 (9)	0.0191 (10)	−0.0040 (9)
C11	0.0394 (12)	0.0309 (11)	0.0314 (11)	−0.0084 (9)	0.0050 (9)	0.0056 (9)
C42	0.0310 (11)	0.0465 (13)	0.0287 (11)	−0.0027 (10)	0.0113 (9)	−0.0041 (9)
C12	0.0467 (14)	0.0279 (11)	0.0485 (14)	−0.0021 (10)	0.0178 (11)	−0.0062 (10)
C35	0.0282 (12)	0.0679 (17)	0.0473 (14)	−0.0169 (11)	0.0176 (11)	−0.0008 (12)
C7	0.0408 (13)	0.0611 (16)	0.0385 (12)	−0.0009 (12)	0.0261 (11)	0.0052 (11)
C21	0.0705 (19)	0.0537 (16)	0.0314 (13)	0.0160 (14)	0.0069 (13)	0.0056 (12)
C14	0.0536 (16)	0.0370 (13)	0.0582 (16)	−0.0176 (12)	0.0096 (13)	−0.0002 (12)

### Geometric parameters (Å, º)

**Table:**

Ag1—O1	2.4125 (19)	C38—H38	0.9500
Ag1—O2	2.4227 (16)	C5—C6	1.384 (3)
Ag1—P1	2.4287 (5)	C5—C4	1.394 (3)
Ag1—P2	2.4570 (5)	C5—H5	0.9500
P1—C8	1.819 (2)	C31—C32	1.388 (3)
P1—C15	1.8234 (19)	C31—H31	0.9500
P1—C1	1.8282 (19)	C4—C7	1.510 (3)
P2—C29	1.8182 (18)	C16—C17	1.382 (3)
P2—C36	1.8209 (19)	C16—H16	0.9500
P2—C22	1.8269 (19)	C9—C10	1.387 (3)
O1—N1	1.249 (3)	C9—H9	0.9500
O2—N1	1.233 (3)	C20—C19	1.386 (3)
C29—C30	1.389 (3)	C20—H20	0.9500
C29—C34	1.397 (3)	C41—H41	0.9500
C2—C3	1.392 (3)	C6—H6	0.9500
C2—C1	1.392 (3)	C32—C33	1.392 (3)
C2—H2	0.9500	C32—C35	1.503 (3)
C25—C26	1.388 (3)	C33—H33	0.9500
C25—C24	1.394 (3)	C28—H28A	0.9800
C25—C28	1.507 (3)	C28—H28B	0.9800
C1—C6	1.397 (3)	C28—H28C	0.9800
C36—C37	1.392 (3)	C19—C18	1.386 (3)
C36—C41	1.392 (3)	C19—H19	0.9500
C26—C27	1.386 (3)	C17—C18	1.390 (3)
C26—H26	0.9500	C17—H17	0.9500
C22—C23	1.393 (3)	C18—C21	1.510 (3)
C22—C27	1.395 (3)	C10—C11	1.381 (3)
C37—C38	1.387 (3)	C10—H10	0.9500
C37—H37	0.9500	C13—C12	1.386 (3)
C27—H27	0.9500	C13—H13	0.9500
C15—C16	1.390 (3)	C11—C12	1.391 (4)
C15—C20	1.394 (3)	C11—C14	1.513 (3)
C23—C24	1.390 (3)	C42—H42A	0.9800
C23—H23	0.9500	C42—H42B	0.9800
C3—C4	1.385 (3)	C42—H42C	0.9800
C3—H3	0.9500	C12—H12	0.9500
C8—C9	1.387 (3)	C35—H35A	0.9800
C8—C13	1.394 (3)	C35—H35B	0.9800
C24—H24	0.9500	C35—H35C	0.9800
C40—C41	1.382 (3)	C7—H7A	0.9800
C40—C39	1.387 (3)	C7—H7B	0.9800
C40—H40	0.9500	C7—H7C	0.9800
C39—C38	1.388 (3)	C21—H21A	0.9800
C39—C42	1.506 (3)	C21—H21B	0.9800
C30—C31	1.387 (3)	C21—H21C	0.9800
C30—H30	0.9500	C14—H14A	0.9800
C34—C33	1.383 (3)	C14—H14B	0.9800
C34—H34	0.9500	C14—H14C	0.9800
O1—N1	1.249 (3)		
			
O1—Ag1—O2	50.80 (7)	C3—C4—C5	118.19 (18)
O1—Ag1—P1	116.26 (6)	C3—C4—C7	120.69 (19)
O2—Ag1—P1	125.62 (4)	C5—C4—C7	121.11 (19)
O1—Ag1—P2	107.68 (7)	C17—C16—C15	120.6 (2)
O2—Ag1—P2	107.83 (4)	C17—C16—H16	119.7
P1—Ag1—P2	124.597 (16)	C15—C16—H16	119.7
C8—P1—C15	104.32 (9)	C8—C9—C10	120.4 (2)
C8—P1—C1	105.61 (9)	C8—C9—H9	119.8
C15—P1—C1	103.18 (9)	C10—C9—H9	119.8
C8—P1—Ag1	113.22 (7)	C19—C20—C15	120.78 (19)
C15—P1—Ag1	119.24 (6)	C19—C20—H20	119.6
C1—P1—Ag1	110.03 (6)	C15—C20—H20	119.6
C29—P2—C36	104.60 (8)	C40—C41—C36	120.68 (18)
C29—P2—C22	105.10 (8)	C40—C41—H41	119.7
C36—P2—C22	103.35 (8)	C36—C41—H41	119.7
C29—P2—Ag1	116.47 (6)	O2—N1—O1	113.4 (2)
C36—P2—Ag1	109.17 (6)	C5—C6—C1	120.37 (19)
C22—P2—Ag1	116.74 (6)	C5—C6—H6	119.8
N1—O2—Ag1	97.86 (14)	C1—C6—H6	119.8
C30—C29—C34	119.00 (17)	C31—C32—C33	118.79 (18)
C30—C29—P2	118.98 (14)	C31—C32—C35	120.3 (2)
C34—C29—P2	122.00 (14)	C33—C32—C35	120.9 (2)
C3—C2—C1	120.60 (18)	C34—C33—C32	120.79 (19)
C3—C2—H2	119.7	C34—C33—H33	119.6
C1—C2—H2	119.7	C32—C33—H33	119.6
C26—C25—C24	117.94 (17)	C25—C28—H28A	109.5
C26—C25—C28	120.83 (18)	C25—C28—H28B	109.5
C24—C25—C28	121.15 (18)	H28A—C28—H28B	109.5
C2—C1—C6	118.49 (17)	C25—C28—H28C	109.5
C2—C1—P1	123.02 (14)	H28A—C28—H28C	109.5
C6—C1—P1	117.95 (15)	H28B—C28—H28C	109.5
C37—C36—C41	118.24 (17)	C20—C19—C18	121.0 (2)
C37—C36—P2	123.18 (14)	C20—C19—H19	119.5
C41—C36—P2	118.52 (14)	C18—C19—H19	119.5
C27—C26—C25	121.38 (17)	C16—C17—C18	121.4 (2)
C27—C26—H26	119.3	C16—C17—H17	119.3
C25—C26—H26	119.3	C18—C17—H17	119.3
C23—C22—C27	118.79 (17)	C19—C18—C17	117.9 (2)
C23—C22—P2	123.59 (14)	C19—C18—C21	121.6 (2)
C27—C22—P2	117.61 (14)	C17—C18—C21	120.5 (2)
C38—C37—C36	120.51 (18)	C11—C10—C9	121.4 (2)
C38—C37—H37	119.7	C11—C10—H10	119.3
C36—C37—H37	119.7	C9—C10—H10	119.3
C26—C27—C22	120.37 (17)	C12—C13—C8	120.2 (2)
C26—C27—H27	119.8	C12—C13—H13	119.9
C22—C27—H27	119.8	C8—C13—H13	119.9
C16—C15—C20	118.18 (18)	C10—C11—C12	117.9 (2)
C16—C15—P1	123.38 (15)	C10—C11—C14	121.1 (2)
C20—C15—P1	118.37 (14)	C12—C11—C14	120.9 (2)
C24—C23—C22	120.16 (17)	C39—C42—H42A	109.5
C24—C23—H23	119.9	C39—C42—H42B	109.5
C22—C23—H23	119.9	H42A—C42—H42B	109.5
C4—C3—C2	121.01 (19)	C39—C42—H42C	109.5
C4—C3—H3	119.5	H42A—C42—H42C	109.5
C2—C3—H3	119.5	H42B—C42—H42C	109.5
C9—C8—C13	118.70 (19)	C13—C12—C11	121.3 (2)
C9—C8—P1	118.16 (15)	C13—C12—H12	119.4
C13—C8—P1	123.03 (16)	C11—C12—H12	119.4
C23—C24—C25	121.30 (18)	C32—C35—H35A	109.5
C23—C24—H24	119.4	C32—C35—H35B	109.5
C25—C24—H24	119.4	H35A—C35—H35B	109.5
C41—C40—C39	121.37 (18)	C32—C35—H35C	109.5
C41—C40—H40	119.3	H35A—C35—H35C	109.5
C39—C40—H40	119.3	H35B—C35—H35C	109.5
C40—C39—C38	117.86 (18)	C4—C7—H7A	109.5
C40—C39—C42	120.99 (19)	C4—C7—H7B	109.5
C38—C39—C42	121.14 (18)	H7A—C7—H7B	109.5
C31—C30—C29	120.40 (18)	C4—C7—H7C	109.5
C31—C30—H30	119.8	H7A—C7—H7C	109.5
C29—C30—H30	119.8	H7B—C7—H7C	109.5
C33—C34—C29	120.26 (19)	C18—C21—H21A	109.5
C33—C34—H34	119.9	C18—C21—H21B	109.5
C29—C34—H34	119.9	H21A—C21—H21B	109.5
N1—O1—Ag1	97.87 (15)	C18—C21—H21C	109.5
C37—C38—C39	121.31 (18)	H21A—C21—H21C	109.5
C37—C38—H38	119.3	H21B—C21—H21C	109.5
C39—C38—H38	119.3	C11—C14—H14A	109.5
C6—C5—C4	121.28 (18)	C11—C14—H14B	109.5
C6—C5—H5	119.4	H14A—C14—H14B	109.5
C4—C5—H5	119.4	C11—C14—H14C	109.5
C30—C31—C32	120.75 (19)	H14A—C14—H14C	109.5
C30—C31—H31	119.6	H14B—C14—H14C	109.5
C32—C31—H31	119.6		

### Hydrogen-bond geometry (Å, º)

**Table:**

*D*—H···*A*	*D*—H	H···*A*	*D*···*A*	*D*—H···*A*
C28—H28*B*···O2^i^	0.98	2.34	3.292 (3)	165
C42—H42*B*···N1^ii^	0.98	2.52	3.491 (4)	170

Symmetry codes: (i) *x*−1/2, −*y*+3/2, *z*−1/2; (ii) *x*, *y*, *z*−1.
